# The Diagnostic Tampon of Schultze

**Published:** 1889-10

**Authors:** 


THE DIAGNOSTIC TAMPON OF SCHULTZE.—In our June number, we published this article complete, having received it as an original communication direct from the author, as shown by the following letter:

HONORED COLLEAGUE— . . . . . I send you to-day an original article, which will appear very soon in the *Centralblatt für Gynäkologie.* I wish the subject upon which it treats—the Diagnosis of the Earlier Stages of Endometritis—to attract the attention of the general practitioner.

It will please me if you will have the article translated and receive it as an original communication in your journal.

Should you not publish it in your own, I beg you will send to that journal for which it is adapted.

With fraternal greeting,

Yours, very sincerely, B. SCHULTZE.

JENA, April 30, 1889.

The article was received in advance proof from the *Centralblatt für Gynäkologie,* and was translated by Dr. John Parmenter. The medical journals are giving excerpts from the paper translated from the *Centralblatt,* but we shall be pleased to furnish it entire to any who may apply.

---

AT THE recent annual meeting of the American Association of Obstetricians and Gynecologists held in Cincinnati, Dr. and Mrs. William H. Taylor tendered the Fellows a most brilliant reception on Wednesday evening, September 18, 1889. An opportunity was thus afforded the visitors to become acquainted with the physicians of the Queen City of the West, and many friendships were then formed or renewed.

Dr. Taylor retires from the presidency of the Association after two years' service, and may well be congratulated upon his successful administration of its affairs.

